# Industrial hemp as an agricultural crop in Ghana

**DOI:** 10.1186/s42238-021-00066-0

**Published:** 2021-04-12

**Authors:** Nana Osei Owusu, Benedict Arthur, Emmanuel Mensah Aboagye

**Affiliations:** Zhongnan University of Economics and Law, 182# Nanhu Avenue, East Lake High-tech Development Zone, Wuhan, 430073 People’s Republic of China

**Keywords:** Cannabis, Marijuana, Agricultural crop, Hemp, Industrial agriculture, Ghana

## Abstract

**Background:**

Cannabis is one of humanity’s oldest crops with several uses, from food to clothing and medicine. It remains one of the most controversial crops whose production, possession, and usage are regulated differently across jurisdictions. Academic research and advocacy have resulted in the redefinition of the legal status of cannabis in several countries. Ghana recently reviewed its laws on cannabis, allowing for the cultivation of industrial hemp. The legislation paves the way for Ghana to benefit from industrial hemp and include it in the agricultural cash crop list. This paper looks at the economic prospects of industrial hemp in the wake of the new law.

**Methods:**

A systematic electronic research was conducted to identify journal articles, reports, news, blogs, and other relevant materials on cannabis, marijuana, and industrial hemp. The electronic search was done primarily on Google, Google Scholar, Bing, and “Baidu Xueshi” to identify cannabis-related publications. The search was expanded beyond Ghana to find other perspectives on cannabis. The search began in January 2020 on Google using search terms like “cannabis in Ghana” and “which countries have legal cannabis.” Materials on history, financial prospects, industrial uses, and legislations on cannabis and industrial hemp were reviewed.

**Results:**

Existing research on cannabis in Ghana has focused on the psychotic effects of cannabis other than its industrial aspects, which has potentials for the economy. Industrial hemp has CBD with no psychotic effects and is very useful in making medicine, paper, and textiles. Ghana has both the land and workforce to produce hemp to feed local industries and the international market.

**Conclusion:**

The new legislation can put Ghana in a position to benefit from the current cannabis industry. Therefore, policymakers should implement a registration regime that would favor local investors and farmers to reduce illegal production. The regulatory framework should establish a well-equipped agency that will supervise production and research into hemp development.

## Background

Cannabis is said to have originated in central Asia and was cultivated mainly in China for several uses (Clarke and Merlin 2013; Ranalli 1999; Ren et al. 2019). It spread from Central Asia through East Asia, South Asia, and Europe (Ranalli 1999). The plant served multiple purposes, including hemp for fabric, rope, animal fodder, cooking oil, medicine, paper, and religious purposes. According to Ranalli (1999), cannabis use is influenced by culture. European, northern, and eastern Asian cultures focused on fiber and seed production. In contrast, Middle Eastern, African, South, and Southern Asian cultures used it primarily as a psychoactive drug and secondarily for seed plant and fiber.

Existing research suggests that cannabis was introduced in Ghana by ex-servicemen who fought in World War II (Bernstein 1999; Mensah and Adu-Gyamfi 2019). The usage was common among ex-servicemen and certain strenuous and dangerous jobs such as stevedores, fishers, prostitutes, criminals, farmers, and night-soil men (Akyeampong 2005). These groups of people are often regarded as low class. However, the usage of cannabis in Ghana has evolved. The substance has become common among all classes, including students in tertiary institutions (Adu-Gyamfi and Brenya 2015) and secondary schools (Adu-Mireku 2003).

The records of the existence and uses of cannabis date back to over 6000 years (Sawler et al. 2015). The plant was a very legal one, and early colonists in Northern America were required to grow it (Deitch 2003; Segal 2014). The *1937 Marihuana Tax Act* effectively outlawed cannabis in the USA by imposing strict regulations and prohibitive taxes that increased the cost of legally trading in cannabis (Johnson 2018). However, there has been a gradual shift, with more states relaxing the laws on cannabis. The Farm Bill 2018 legalized industrial hemp production at the federal level. Cannabis has been a popular plant among the Chinese. Tourists who visited the Yunnan province due to the “open door policy” began secretly smoking the flowers for the psychoactive effect. As a solution to preventing the abuse and ensuring the locals still grew hemp for fiber, the local government introduced industrial hemp. Notwithstanding China being a major world producer of industrial hemp, the regulations vary in different provinces. Several other countries have revisited the legal status of cannabis as a result of developments in cannabidiols (medical cannabis).

Despite cannabis being illegal in most African countries, current data suggest extensive usage in the region. According to the cannabis use perception index, cannabis use increased between 2010 and 2016. The increase was significant in Asian and African countries, followed by countries in the Americas and Europe (World Drug Report 2018 (Sales No. E.18.XI.9) n.d.). With annual prevalence rates of 13.2%, the continent has one of the highest consumption rates in the world (Prohibition Partners 2019). In 2016, Africa recorded 17% of total seizes of cannabis in the world (World Drug Report 2018 (Sales No. E.18.XI.9) n.d.). However, countries like Lesotho, South Africa, Zimbabwe, Zambia, and Malawi have taken the lead by legalizing cannabis to reap their medical and economic benefits.

Kilmer and Pacula (2017) identified four thematic areas that cannabis laws address. These are purpose—medical and recreational purposes; producer—who is granted protection under the law for large-scale production; purchase—who can purchase cannabis and its related products; and products—what kinds of cannabis-related products are permitted. One of the major arguments for the legalization of recreational use is that it allows the government to benefit from the use of cannabis through legalization and reduce the burden of drug enforcement agencies (Lynn 2016). However, recreational use was not the focus of this paper.

**Fig. 1:**
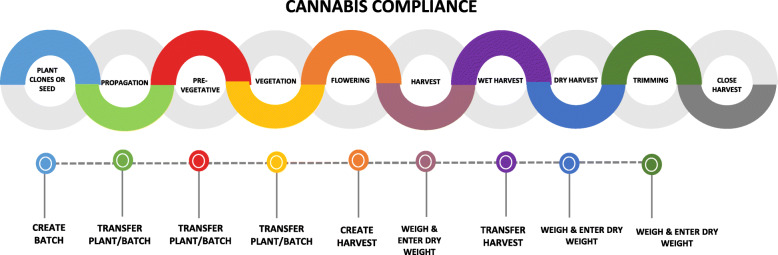
Cannabis compliance in Lesotho. Source: Medikingdom “Production” https://www.medikingdom.com/

A country like South Africa allows citizens to grow specific amounts for personal medical and non-medical purposes. Other countries adopt the legalization and commercialization approach to cannabis legislation. This allows for production under license for commercial and research purposes. In this approach, *Cannabis sativa* L cultivated as industrial hemp can be produced and its related products extracted by a company under a license. The license issuing authority strictly monitors the production and supply chain. Figure 1 shows the compliance regime that regulates the production of cannabis by *Medikingdom* in Lesotho.

OGD (1995), as cited in Bernstein (1999), reported Ghana to be a significant producer of high-quality cannabis second to Nigeria in West Africa. Ghana, Nigeria, and Eswatini were identified as a primary source and trafficking points for cannabis in Africa (World Drug Report 2018 (Sales No. E.18.XI.9) n.d.). The report also noted that most of the cannabis produced in Ghana was for export. These reports suggest that the country has the potential to produce for the legal cannabis market. Ghana recently passed the Narcotics Control Commission Act, 2020 (Act 1019) (2020). This act was to repeal *the Narcotic Drugs (Control, Enforcement, and Sanction) Act, 1990, PNDC Law 236,* which criminalized possession or importation of narcotic substance (cannabis inclusive). A violation of the law was punishable by up to 10 years of imprisonment. Also, Act 1019 decriminalizes the use of cannabis for commercial and health purposes.

Although several researches have explored the use of cannabis in Ghana, there has been little or no documentation on cannabis as an industrial agricultural crop. This has been so mainly due to the effect and abuse associated with the use of marijuana in Ghana. As a result, more theoretical and empirical researches are needed to ascertain the use of cannabis as an agricultural crop in Ghana. In this regard, this study contributes to the literature by exploring hemp as an industrial agriculture crop in Ghana. Additionally, to the best of the authors’ knowledge, no study has been conducted on hemp as an industrial agricultural crop in Ghana following the enactment of Act 1019.

## Methods

The first electronic search was *Ghana marijuana* and *Ghana cannabis* on Google and Google Scholar. The commonly used local names of marijuana or cannabis were also included in the search to generate more results peculiar to the Ghanaian situation. Local jargons such as “ntampi,” “abonsam tawa” (which means devil’s tobacco), and “wee Ghana” were also searched on Google and Google Scholar. The search method is similar to that of Kruger et al. (2020) and Zembroski (2011). The search terms on Google were to identify general information from non-academic publications, blogs, news articles to academic publications, and books about cannabis. The search on Google Scholar was to identify academic publications on cannabis in Ghana.

**Fig. 2:**
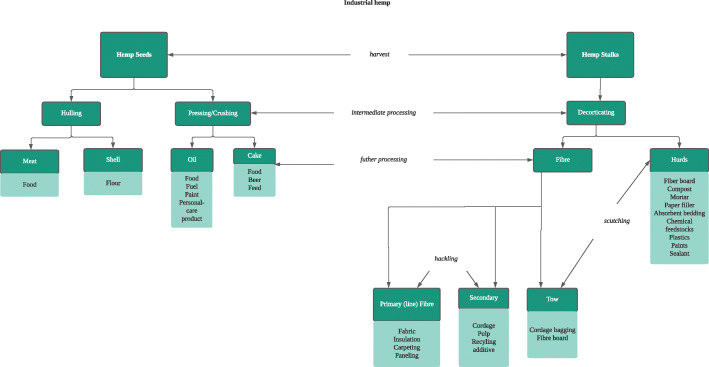
Search sequence. Source: authors’ construct

The search was expanded to get other relevant information on cannabis. The terms *marijuana*, *cannabis*, *uses of cannabis*, *importance of marijuana*, *cannabis industry*, *countries with legal cannabis*, *African countries with legal cannabis*, and *industrial hemp* were searched on Google, Google Scholar, Bing, and “Baidu Xueshi” (a Chinese scholarly database). The search query was narrowed to the following thematic areas *medicine*, *cannabis and cancer treatment*, *hemp as textiles*, *hemp as paper*, and *hemp as oil and food* (Fig. 2)*.* To identify the contribution of agriculture to Ghana’s economy, the terms *agriculture to Ghana’s GDP* and *planting for food and jobs* were searched on all selected search engines.

The obtained search results were grouped into financial prospects (reviewed BA), uses, legislation, and policy (reviewed by NOO and EMA). Out of the forty-three (42) articles cited, twenty-five (25) were published between 2015 and 2020. Twelve of the 42 were published between 2000 and 2014 and 5 before 2000.

The help of Mr. Boakye-Ansah was solicited to retrieve the hard copy of Act 1019 from the Parliamentary library. Mr. Nyarko was also consulted to verify some of the news publications on cannabis in Ghana.

## Results

The term “cannabis in Ghana” on Google Scholar produced 19 journal articles. Most articles focused on the abuse of cannabis or marijuana, their psychoactive effect, and illicit drug enforcement. However, three articles out of the 19 studied other aspects of cannabis that were more relevant to this study’s objective (see Table 1).

**Table 1:** Results from the search term “cannabis in Ghana”

SN	Author	Theme
1	Mensah and Adu-Gyamfi (2019)	Pharmacological uses of cannabis
2	Agyapong (2019)	Chemical content of illegally cultivated cannabis
3	Akyeampong (2005)	Historical overview of drug trafficking

Source: authors’ construct

The search terms *Ghana marijuana* and *Ghana cannabis* generated 4,660,000 and 4,950,000 results, respectively, on Google. Most of the results were news articles and social commentary that reported the legalization of cannabis in Ghana. The search on Google using the local jargon, for instance, “abonsam tawa,” generated 1200 results on Google and 62 on Bing. Most of the results were articles on people arrested for marijuana possession or cannabis and blogs on advocating for the legalization of cannabis (Fig. 2). Search query from “ntampi” generated more results, which were less irrelevant to the topic’s objective.

The information on industrial uses and comparative legislations of cannabis was identified in search terms, which excluded Ghana. News articles, blogs, or non-academic articles on cannabis and hemp that provided the author’s information (at least their first name) were considered.

In Ghana, before the enactment of Act 1019, all cannabis species were classified as a narcotic substance, and unlawful possession Sections 1, 2, and 5 of PDNC Law 236 stipulated that;

anyone who wants to import or possess any narcotic substance had to obtain a license, which had to be granted by the Minister for Health. Also, anyone who imports a narcotic drug is supposed to submit the details to the Pharmacy council. Possession and usage of narcotic substances, including cannabis, even for medical purposes without following due process, are unlawful and punishable by up to 10 years of imprisonment. [emphasis added]

The Ministry of Health (MoH) was supposed to set up a licensing regime, but it never did until the PNDC Law 236 was repealed. The lack of a regulatory framework encouraged the illegal possession of cannabis and its related products in Ghana.

Ghana shares a common cannabis legislation history with Zambia. In Zambia, the status of medical cannabis was in contradiction. The production of cannabis was technically legalized under the Narcotic Drugs and Psychotropic Substances Act Cap 96. However, cultivation was only allowed with a license from the Health Minister—none had been granted thus far. In 2019, the Zambian government decided to legalize cannabis cultivation when the government decided to export cannabis to boost the economy (Mfula 2019).

Act 1019 provides a new status for cannabis and allows for the production of industrial hemp (which has THC > 0.3%). Section 43 of Act 1019 titled *Special provision relating to cannabis* of reads:

1. … the Minister, on the recommendation of the Commission, may grant a license for the cultivation of cannabis, which has not more than 0.3% THC content on a dry weight basis for the industrial purposes for obtaining fiber or seed or for medicinal purposes.
2. For the avoidance of doubt, a license granted under subsection (1) shall not be for the cultivation of cannabis for recreational use.

Ghana adopts the legalization and commercialization approach for cannabis regulation. This is evident in the strain of cannabis that can be cultivated under license. The legislation allows for industrial and medical purposes only. However, others adopt legislations that do not only allow for commercialization but also for recreational purposes.

**Fig. 3:**
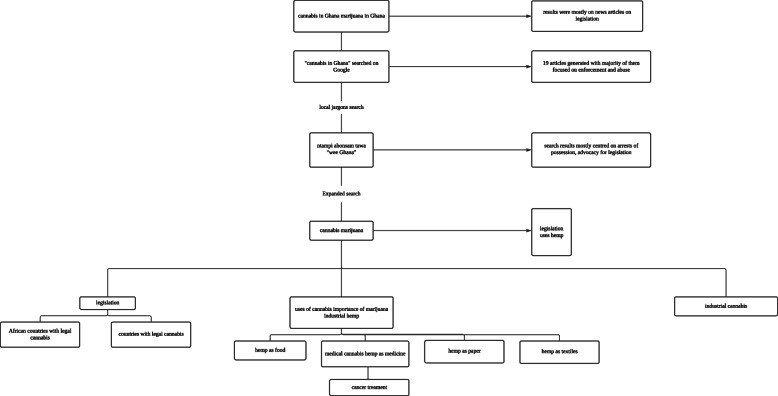
Industrial hemp production. Source: adapted from Kraenzel et al. (1998)

Industrial hemp has a wide range of applications in the health, construction, fashion, energy production, and manufacturing sectors. Industrial hemp is also an environmentally friendly crop and suitable for crop rotation. Some of the applications of industrial hemp might not be currently feasible in Ghana as a result of the current state of technology. For instance, it is currently not feasible to use industrial hemp as biofuel or in construction in Ghana. Nevertheless, the country can cultivate hemp as a raw material for export and make some semi-finished products to feed the local industry. Figure 3 shows the various products that can be derived from hemp at harvest to post-harvest processing.

### Medicine

In 2018, the US Food and Drug Agency approved Epidiolex, the first prescription medication to contain CBD. Epidiolex is used for treating rare, difficult-to-control epilepsy (Holland 2020). CBD has also been useful for managing chemotherapy side effects. CBD has been effective in treating pain, relieving nausea, and stimulating appetite among cancer patients (Davis 2016; Javid et al. 2016). Current research also shows that CBD can prevent prostate cancer (Sharma et al. 2018), while Shrivastava et al. (2011) also concluded that CBD prevents breast cancer cells’ growth. Ghana recorded 912 and 2260 prostate and breast cancer incidents, respectively (World Health Organization (WHO). 2014). In 2020, the number of prostate and breast cancer incidences increased to 2129 and 4482 (GLOBOCAN 2020). With current growing concerns about the new cases of breast and prostate cancer in Ghana, there is a need to explore CDB use. CBD has several other pharmaceutical potentials that the nation can benefit from. The legalization and commercialization present a new opportunity for the Ghanaian pharmaceutical industry to research into CBD and possibly produce CBD-related drugs for the local market. Also, herbal medicine producers can explore the medical potential of hemp. Hemp is also used in supplements and medicinal and therapeutic products.

### Hemp for textiles and fiber

For thousands of years, hemp was traditionally used as clothing. Synthetic fibers and cotton currently meet the bulk of textile fibers’ demands. Fiber hemp may be an alternative to cotton and synthetic fibers as a raw material for textile (Westerhuis 2016). Hemp is lightweight, with three-time the tensile strength of cotton. Clothes made from industrial hemp have many good qualities. They are antibacterial, anti-static, and warmer (Brady 2003). Also, hemp fabrics dry faster and provide excellent protection from UV rays. The porous nature of hemp fabric makes it more absorbent. Compared to cotton, hemp is environmentally friendly. Brady (2003) noted that industrial hemp requires little or no pesticides, herbicides, or fertilizers. Considering the climatic conditions in Ghana, clothes made from hemp fiber are more suitable. Hemp also serves as a rotational crop and can be intercropped with other crops. It protects the surface of the soil and prevents erosion. Also, hemp fiber is recommended for the making of sound absorption fiber because it exhibits a robust acoustic performance (Liao et al. 2020).

The textile industry in Ghana plays a major in the manufacturing industry. The sector was very vibrant and employed over 25,000 workers (Quartey 2006). Finished textiles made by the Ghanaian industries were in high demand on the local market because they were mainly used to produce and design traditional garments such as “Kaba” and other attractive gears (Bruce-Amartey Jr et al. 2014). The textile industry has dwindled in the past couple of years. The introduction of textiles made from hemp, which is gradually gaining popularity in the modern fashion industry, will likely increase the prospects of the struggling textile industry. Therefore, legalization and commercialization provide new opportunities for farmers and hope for the textile industry.

### Hemp for paper

Ghana imported US$177.38 worth of paper and paperboard, articles of pulp, paper, and board.

The long fibers found in industrial hemp produce high-quality paper for magazines, books, and stationery, while the shorter fibers are suitable materials for tissue, newspaper, and packaging materials. Compared with wood pulp, industrial hemp paper resists decomposition and does not discolor (brown or yellow) with age (lmartin 2019). Kimberly Clark, a Fortune 500 company, has an industrial hemp-paper mill in France, which makes paper for Bibles and cigarettes due to its durability and failure to discolor with age (Brown 1998). There has been promising research on the use of hemp for specialty papers. Products in the specialty paper markets include teabag, cigarette papers, currency, and filter papers. Hurter Consult, Inc., a Canadian company, was hired to conduct a pre-feasibility study for Prairie Pulp and Paper Co. for the use of flax straw and industrial hemp to produce either pulp or un-coated printing and writing paper (Brady 2003).

### Oil as a foodstuff

Oil extracted from hempseeds can be used for cooking. The hemp seed oil has high concentrations of polyunsaturated essential fatty acids, alpha-linolenic acid-omega, linoleic-omega 6, and gamma-linoleic acid. These compounds are an alternative to saturated fats and have a variety of health benefits, which include the prevention of the development of coronary heart diseases. The seeds for oil have to be sterilized in a process that does not involve excessive heat.

Hemp seed can also be used as foodstuff for both animals and humans. Hemp is used in birdseed mixes. After oil extraction, the crushed seeds are a source of high protein content, making hemp a valuable livestock feed. The seeds also have traces of vitamin A. After oil extraction, the crushed seeds can be processed to flour for the making of bread, cake, pasta, and biscuits.

## Discussion

Prohibition Partners (2019) estimates Ghana’s medicinal cannabis market value at 0.38(US$m) and recreational market value of 326 (US$m) by 2023. The legalization and commercialization of industrial hemp create new foreign direct investments (FDI) for the country. The Ghana Beyond Aid agenda aims to transform the economy from foreign aid-dependent to a self-reliant and investment-driven economy. This has led to aggressive policies by the government and pragmatic steps that have seen many successes with some automobile giants like Volkswagen and Nissan setting up assembly plants in Ghana. The cannabis industry is a very lucrative one. There are currently 25,000 industrial hemp-based products globally. This ranges from automotive parts, furniture, textiles, foods, beauty products, and construction supplies (Carpenter 2018). The US legal market for CBD, extracted from industrial hemp, is estimated to be $20 billion (Giammona 2018). Brightfield Group’s analysis of the Canadian cannabis industry predicts it to be worth 5 billion by 2021. A similar analysis by New Frontier Data predicts that Canadian cannabis will be worth 2025 by 2025 (Subramaniam 2020). US hemp imports for 2017 were $67.3m, with 90% of these coming from Canada (Johnson 2018). All these economies need an external supply of raw materials or semi-finished cannabis products for their market. Lethoso saw an FDI inflow of $13.13m in its cannabis industry (Prohibition Partners 2019).

Ghana has the climatic and soil conditions suitable for the production of industrial hemp. Despite the technological challenges faced by farmers in Ghana, agriculture contributes significantly to the GDP and employment. In 2014, agriculture contributed about 23.6% to GDP, 22.8% in 2015, and increased to 23% in 2016. In 2017 and 2018, it contributes 22% and 20.5%, respectively. The Planting for Food and Jobs (PFJ) program launched in 2017 was to modernize agriculture to create more jobs and reduce poverty (Government of Ghana 2019). The program created 11,270 temporary and 2700 permanent graduate jobs. Also, ten tertiary institutions participated in the PFJ. These tertiary institutions are in a position to lead cannabis development research for production maximization. This suggests that with the appropriate policies in place, agriculture can contribute more to Ghana’s development. Cannabis is reported to have been a source of income for cocoa farmers during global price falls. Cocoa farmers were said to have intercropped cocoa with cannabis (Akyeampong 2005; Bernstein 1999). A police investigative report in 2001 indicated that some Ghanaian farmers found cannabis farming as more lucrative than maize farming Salifu (2001) as incited in Akyeampong (2005).

## Conclusion

This paper suggests that the new legislation can have a positive impact on the Ghanaian economy. This conclusion differs from Mensah and Adu-Gyamfi (2019) and Osei (2016) primary arguments against the legalization of cannabis. Their arguments were mainly based on the psychoactive compounds on cannabis.

Although the new legislation has economic prospects for the country, there is a need to consider the industry entry requirement carefully. Ghana should draw lessons from current licensing regimes in Africa, which seems to be a disadvantage for local farmers and investors. Licensing fees should be flexible and a framework that will allow local entrepreneurs to have a foothold in the hemp industry. For instance, in Lesotho, only big corporations can afford the heft $37,000 license fee and small-scale farmers are still growing hemp illegally. Evidence from Malawi and Eswatini suggests the African cannabis industry is dominated by foreign countries (Prohibition Partners 2019). There is also a need for strict supervision of the cultivation to prevent the abuse of license granted.

Several extraction industries can be created from industrial hemp production; hence, the licensing regime needs to favor local investors and farmers to boost the Ghanaian manufacturing sector. However, Ghana can partner with institutions in China, Canada, and the USA that have conducted several kinds of research into the production of hemp so that Ghana will be better equipped to regulate and benefit from hemp production.

## Data Availability

Not applicable

## Abbreviations

CBD: Cannabidiol
MoH: Ministry of Health
ODG: Observatoire Geopolitique de Drogues
PFJ: Planting for Food and Jobs
PNDC: Provincial National Defence Council
THC: Tetrahydrocannabinol
WHO: World Health Organization
