# Correction to: Industrial hemp as an agricultural crop in Ghana

**DOI:** 10.1186/s42238-021-00076-y

**Published:** 2021-06-05

**Authors:** Nana Osei Owusu, Benedict Arthur, Emmanuel Mensah Aboagye

**Affiliations:** Zhongnan University of Economics and Law, 182# Nanhu Avenue, East Lake High-tech Development Zone, Wuhan, 430073 People’s Republic of China

**Correction to: Journal of Cannabis Research 3, 9(2021)**

**https://doi.org/10.1186/s42238-021-00066-0**

Following the publication of the original article (Owusu et al. 2021), it was noted that Figs. 2 and 3 were switched.

The original article has been updated to correct the figures.
